# Bioinformatics and Data Management Support for Environmental Genomics

**DOI:** 10.1371/journal.pbio.0030297

**Published:** 2005-08-16

**Authors:** Dawn Field, Bela Tiwari, Jason Snape

## Abstract

The UK Natural Environment Research Council has funded the creation of a dedicated bioinformatics centre as part of a £26m Environmental Genomics initiative.

As concerns over pollution and climate change increase, understanding the impact of environmental change on living organisms is coming to the fore as never before. Research in the area of environmental genomics, through the application of genomic technologies, is shedding light on fundamental processes by which organisms evolve and adapt to both the biotic and abiotic aspects of their environments. Understanding how organisms perceive and react to changes in their environments also has many direct applications, for example, in the field of bioremediation.

The United Kingdom Natural Environment Research Council (NERC) has recently invested over £26 million in this area under the Environmental Genomics and Post-Genomics and Proteomics Science Programmes. As part of the Environmental Genomics initiative, NERC funded the creation of a dedicated bioinformatics centre with a remit to provide bioinformatics consultation, training, and data management.

The Environmental Genomics Thematic Programme Data Centre (EGTDC) was established in 2002 and works to develop and implement bioinformatics and data management solutions for environmental genomics researchers. The EGTDC team of bioinformaticians and data managers work together to develop a variety of open-source projects including Bio-Linux, a Linux computing platform customized for bioinformatics research, Partigene, an EST analysis pipeline, and Maxd, a transcriptomics software suite that specializes in aiding users in annotating experiments to MIAME standards. The EGTDC also presents courses on a range of topics, has developed an extensive set of Web-based documents, and aims to make all of the resources it develops available to the wider public.

The EGTDC is particularly interested in the use and development of data standards in this area and has developed an “ENV” extension to MIAME to capture information about microarray experiments relevant to environmental samples. This activity is now formally recognized by the Microarray Gene Expression Data Society.

Through the combined provisioning of computers, software, appropriate data standards, and bioinformatics consultation, the EGTDC aims to help researchers more easily collect, store, and interpret their genomic data (Figure 1). To maximize the collective value of this data, the EGTDC has recently developed a public data catalogue (Figure 2). Discovery-level metadata are stored along with accession numbers for data held in public databases (dbEST, ArrayExpress, EMBL, etc). Therefore, the data catalogue is a way for users to “spider out” to the digital datasets that may be held in primary public databases and in specialized databases generated by the environmental genomics research community, the EGTDC, or individual researchers. The catalogue currently holds information for 28 environmental genomic grants and a total of 314 data holdings of a variety of types, including “omic” data (genomics, arrays, and proteomics data), single nucleotide polymorphisms, microsatellites, genetic maps, libraries, publications, and other documents, such as protocols and phenotypic data. This catalogue can be searched by keyword, data type, and a variety of other fields and provides accession numbers that can be cited in publications or linked to (accessed) directly by URL. Anyone interested in submitting information on existing or future datasets can contact the EGTDC helpdesk at E-mail: helpdesk@envgen.nox.ac.uk.

**Figure 1:**
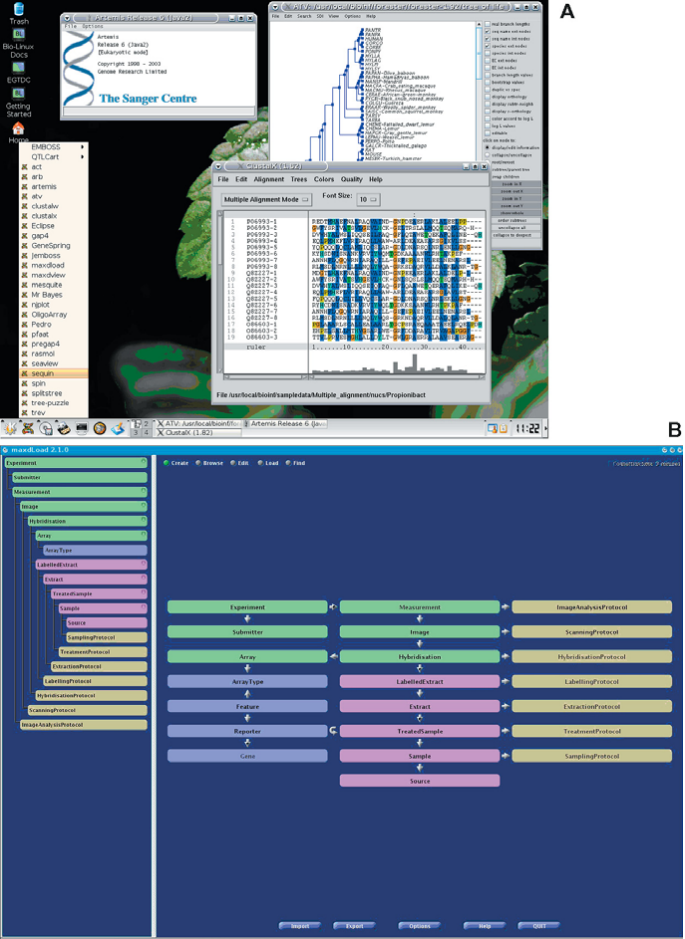
The Bio-Linux Computing Platform (A) Bio-Linux is a Linux distribution customized to be user-friendly that contains approximately 60 popular bioinformatics packages. The Bio-Linux system is freely available, though researchers not supported by the EGTDC must provide their own hardware. Software developed at the EGTDC is included on the Bio-Linux system, making it easy for researchers not well versed in computing to try out these packages. The graphical menus and wide range of bioinformatics software installed on Bio-Linux makes it an ideal system for all levels of users, including beginners, power users, and developers. (B) MaxdLoad2, one of the tools installed on Bio-Linux, provides an intuitive interface to annotate experiments to MIAME standards. When completed, the experiment annotation can be exported in MAGE-ML for submission to ArrayExpress. MaxdLoad2 has been recently engineered to capture data from the “ENV” extension to MIAME.

**Figure 2:**
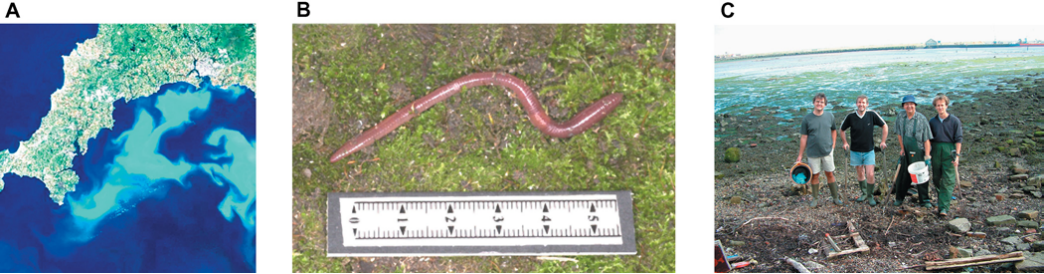
The EGTDC Data Catalogue The EGTDC data catalogue currently contains descriptions of 28 environmental genomic grants, each of which can be viewed by its accession number (http://envgen.nox.ac.uk/). Projects include the following studies. (A) How viral infections affect natural phenomena like marine algal blooms (egcat:000010). A virus-infected bloom of the microalga Emiliania huxleyi in the English Channel. Up to 50 million viruses per millilitre were observed in this bloom. (B) The use of earthworms as sentinels of heavy metal pollution in soils (egcat:000024). (C) The genes responsible for circadian and tidal rhythmicity in marine worms (egcat:000029). The “Worm Team” from left: Cas Kramer, Thierry Bailhache, Peter Olive, and Kim Last, ready for the collection of king ragworms in the Blyth Estuary. ([A] Image: Remote Sensing Data Analysis Service/Plymouth Marine Laboratory; [B] Image: Dr. A. John Morgan; [C] Image: Kim Last)

The EGTDC is currently expanding its remit to include future support for proteomics and metabolomics data management and integration of solutions in a systems biology context, with funding from the NERC Post-Genomics and Proteomics Science Programme. The EGTDC is currently funded until early 2009 and hopes to continue, in collaboration with others, to improve the range of bioinformatics tools and databases available for researchers working in environmental genomics. Its vision for the future includes the ability to integrate its genomic holdings with biological and environmental datasets held across the NERC and beyond. The EGTDC expects to change its name to the NERC Environmental Bioinformatics Centre in the near future, but its main remit will remain the collection, curation, and management of genomic data of environmental relevance.

More information can be found on the EGTDC Web site at http://envgen.nox.ac.uk.

## Abbreviations

EGTDC: Environmental Genomics Thematic Programme Data Centre
NERC: United Kingdom Natural Environment Research Council

